# Medical Department of the University of Texas: Present Status

**Published:** 1889-11

**Authors:** 


### EDITORIAL DEPARTMENT.

F. E. DANIEL, M. D., EDITOR AND PUBLISHER.

THIS JOURNAL, by unanimous vote of the members, was made the Official Organ of the Austin District Medical Society.

COLLABORATORS:

*Dr. R. M. Swearingen, Austin.*
*Prof. B. E. Hadra, M. D., Galveston.*
*Prof. Geo. Cupples, M. D., San Antonio.*
*Dr. T. C. Osborn, Cleburne, Texas.*
*Dr E. J. Doering, Chicago.*
*Dr. E. J. Beall, Fort Worth.*
*Dr Odo Betz, Germany.*
*Dr. J. W. McLaughlin, Austin.*
*Dr. T. J. Tyner, Austin.*
*Prof. J. F. Y. Paine, M. D., Galveston.*
*Dr. R. H. L. Bibb, Mexico.*
*Dr. T. J. Bennett. Austin.*
*Dr. Bat Smith, Wharton.*
*Dr. E. Meierhof, New York.*
*Prof. W. B. Rogers, M. D., Memphis, Tenn.*

### THE MEDICAL DEPARTMENT OF THE UNIVERSITY OF TEXAS. PRESENT STATUS.

As the medical profession of Texas and the public are deeply interested in all that pertains to the advancement of medicine in Texas, and look with anxious eyes to the time when our own State medical school shall be in successful operation, we have gleaned the following facts from the Board of Regents, and present them to our readers.

It will be remembered that Mr. John Sealey, in his will, donated $50,000, with which to build a hospital for the poor of Galveston city and county, making his brother, Mr. Geo. Sealey, executor of the will, with discretion as to details. The City Council of Galveston, through representatives in the Twentieth Legislature, offered to donate this hospital and the block of ground on which it is situated, to the State for the benefit of the medical department of the State University, on condition that the Legislature would donate $50,000, with which to construct a college building. The proposition was accepted, and the appropriation was made. The hospital is now in operation, and is the property of the medical department of the University of

Texas. It has all the modern improvements and conveniences of a hospital, and is a splendid property. The beautiful structure occupies an elevated and commanding site upon the beach.

It was found, upon investigation by the Regents, that $50,000 donated by the Legislature, was inadequate to the construction of such buildings as they deemed advisable for the medical college, and that the block occupied by the Sealy hospital was not large enough for both buildings; and the Regents declined to build until further means could be provided; accordingly the City Council of Galveston proposed to the Twenty-first Legislature to donate $25,000 more, if the State would appropriate a like amount, with which to erect the college building. This proposition was also promptly met, and with this additional $50,000 the Regents felt safe in going ahead. Accordingly they purchased a most desirable site, the block adjoining the hospital; advertised for plans, etc., agreed upon one—one which had been submitted by one of the ablest architects—and then advertised for bids for the construction of the building in accordance therewith. The building is to be of pressed brick, with facades, relieved of monotony by ornamental trimming. It is to be 200 feet long, by 140 feet deep, and will cost, complete, $75,000. It is to be finished in one year from date of contract. The architect is Mr. A. J. Clayton, of Galveston, and the contractor is Mr. A. Braumbach, of Houston. The hospital just completed is leased to the City of Galveston for a period of years with a reserve of certain clinical privileges for the benefit of the college for a period of twenty-five years.

The building wlll be modelled and constructed in accordance with the latest and approved plans for such buildings, and will contain every convenience and appurtenance for comfort as well as for the purposes of medical instruction,—and Texas will have a medical college second to none in point of beauty of structure. The Hon. President of the Board of Regents, Dr. T. D. Wooten, to whom we are indebted for most of the above information, says in a note to us:

"When the buildings are completed, and the grounds enclosed, improved and ornamented, the whole affair will be both grand and beautiful, and in keeping with the charms of the Island City, and should elicit the interest and pride of every Texas doctor, that it may become a great medical center of learning, a blessing to humanity, a factor in the civilization progress and glory of Texas."

